# New Application Field of Polyethylene Oxide: PEO Nanofibers as Epoxy Toughener for Effective CFRP Delamination Resistance Improvement

**DOI:** 10.1021/acsomega.2c01189

**Published:** 2022-06-24

**Authors:** Emanuele Maccaferri, Jacopo Ortolani, Laura Mazzocchetti, Tiziana Benelli, Tommaso Maria Brugo, Andrea Zucchelli, Loris Giorgini

**Affiliations:** † Department of Industrial Chemistry “Toso Montanari”, University of Bologna, Viale Risorgimento 4, Bologna 40136, Italy; ‡ Interdepartmental Center for Industrial Research on Advanced Applications in Mechanical Engineering and Materials Technology, CIRI-MAM, University of Bologna, Viale Risorgimento 2, Bologna 40136, Italy; § Department of Industrial Engineering, University of Bologna, Viale Risorgimento 2, Bologna 40136, Italy

## Abstract

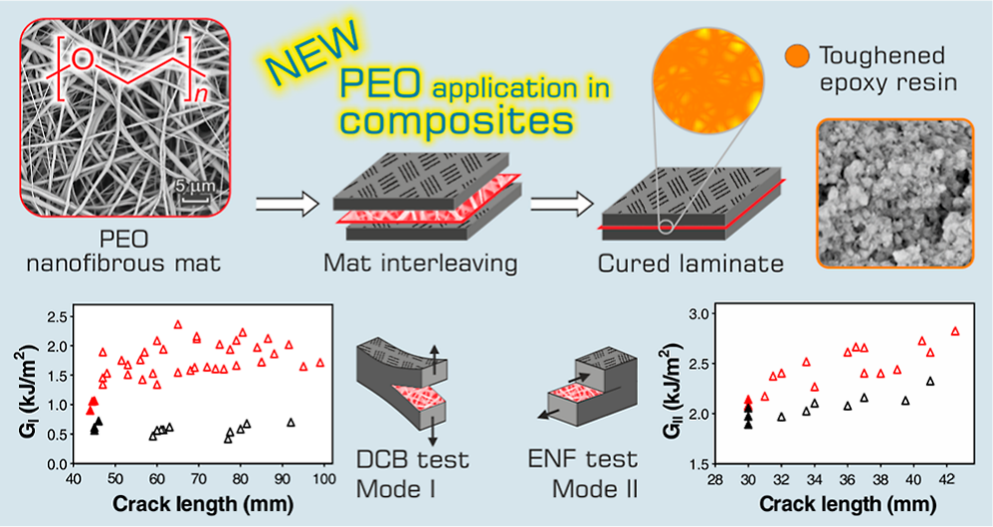

Delamination is the most severe weakness affecting all composite materials with a laminar structure. Nanofibrous mat interleaving is a smart way to increase the interlaminar fracture toughness: the use of thermoplastic polymers, such as poly(ε-caprolactone) and polyamides (Nylons), as nonwovens is common and well established. Here, electrospun polyethylene oxide (PEO) nanofibers are proposed as reinforcing layers for hindering delamination in epoxy-based carbon fiber-reinforced polymer (CFRP) laminates. While PEO nanofibers are well known and successfully applied in medicine and healthcare, to date, their use as composite tougheners is undiscovered, resulting in the first investigation in this application field. The PEO-modified CFRP laminate shows a significant improvement in the interlaminar fracture toughness under Mode I loading: +60% and +221% in *G*_I,C_ and *G*_I,R_, respectively. The high matrix toughening is confirmed by the crack path analysis, showing multiple crack planes, and by the delamination surfaces, revealing that extensive phase separation phenomena occur. Under Mode II loading, the *G*_II_ enhancement is almost 20%. Despite a widespread phase separation occurring upon composite curing, washings in water do not affect the surface delamination morphology, suggesting a sufficient humidity resistance of the PEO-modified laminate. Moreover, it almost maintains both the original stiffness and glass transition temperature (*T*_g_), as assessed via three-point bending and dynamic mechanical analysis tests. The achieved results pave the way for using PEO nanofibrous membranes as a new effective solution for hindering delamination in epoxy-based composite laminates.

## 1 Introduction

Delamination severely limits the widespread application of high-performance fiber-reinforced polymer (FRP) laminates, hampering the replacement of metals in specific fields and the benefits of the lightweight composite.^1,2^ In the years, much effort has been made to avoid the catastrophic consequences of delamination, both by increasing the interlaminar properties (fracture toughness^3−9^ and shear strength^10−13^) and by using integrated sensors able to promptly detect composite damages before complete component failure.^14−19^ The first approach is undoubtedly the most applied due to the high cost and technological issues associated with the implementation of sensors.

Matrix toughening of brittle thermosetting epoxy resins by adding thermoplastic or rubber polymers is a common practice for contrasting the formation and propagation of microcracks.^20−23^ Usually, the modification affects the resin bulk, potentially lowering thermal and mechanical composite properties, such as the glass transition temperature (*T*_g_) and elastic modulus and strength.^24^ Thus, localized interlaminar toughening would be preferred for retaining the overall outstanding properties of high-performance FRP laminates.^25^

Nanofibrous mat interleaving between a prepreg laminate is one of the most effective and convenient ways to contrast delamination,^26^ thanks to the balanced equilibrium between toughening and mechanical property retention. A wide range of thermoplastic polymers have been tested as nonwoven interleaves for increasing interlaminar fracture toughness. Most of them are polyamides (mainly Nylon 6 and Nylon 66) and the polyester poly(ε-caprolactone) (PCL).^3,4,27−35^ Specialty polymers, such as polyvinylidene fluoride (PVDF), and aromatic ones, such as polysulfone (PSU) and polyetherimide (PEI), have also been explored.^3,36,37^ Recently, new elastomeric nanofibers based on nitrile butadiene rubber (NBR) were developed^25,38^ and used for increasing the interlaminar fracture toughness of epoxy-based carbon FRP (CFRP) laminates^25,39,40^ as well as for enhancing their damping.^40,41^ These nanofibers enhance the energy release rate (*G*) up to +480%,^39^ an enhancement higher than the one usually achieved by interleaving conventional thermoplastic membranes, whose improvement is no more than 150% in general.^3,4,27−35^

The nanofibers can act against delamination either by the so-called “bridging” mechanism or by matrix toughening^34^ depending on the nanofiber polymer thermal properties. In the first case, the three-dimensional nanofibrous structure, still present upon composite curing, helps keep adjacent laminates together, hampering the delamination (e.g., Nylons, PVDF, PSU, and PEI). By contrast, in matrix toughening, the polymer melts or “fluidizes” and mixes with the resin before its gel-point: the latter thus becomes less fragile due to plasticization phenomena (e.g., PCL and uncrosslinked NBR^39,40^). In both cases, the energy required for the crack propagation increases, making the delamination more difficult to occur.

Researchers have made a great effort to enhance the toughening effect of well-established polymeric nanofibers by adding nano-reinforcements, such as carbon nanotubes,^27,42,43^ or by combining different polymers, for example, producing core–shell nanofibers,^33,35^ polymer-impregnated nanofibers,^4,44^ and blended ones, such as the above-mentioned rubbery nanofibers.^25,39,40^ However, searching for new uses of “basic” and well-established materials is equally important for reducing costs and complexity of manufacturing advanced materials such as nanofibers with integrated high-performance nano-reinforcements or core–shell ones.

Here, the authors present a new application of the well-known polyethylene oxide (PEO) nanofibers as nonwoven mats for hindering delamination in epoxy-based CFRP laminates. The use of PEO for modifying composite materials is unusual and, to date, undiscovered. While only three studies regarding the addition of PEO copolymers (not in fiber form) as tougheners for bulk resins (not in fiber-reinforced composite laminates) are found in the literature,^45−47^ the use of the PEO homopolymer as a localized resin modifier in CFRP laminates is still not reported. Indeed, thanks to its biocompatibility, PEO is one of the most preferred polymers for producing nanofibrous membranes for use in biomedical and healthcare applications, such as drug delivery, wound healing, and scaffolds.^48−54^ Consequently, the use of PEO nanofibers for composite modification represents a completely different application field than the current ones. For these reasons, the performance of PEO-modified laminates is undiscovered, and it needs proper investigation. For the same reason, any comparison with literature is not viable: a rough comparison can be done only considering the PEO behavior and the one of the similar polymers under the CFRP curing conditions.

The polyether PEO has almost the same thermal properties (*T*_g_ and melting temperature) as the widely used polyester PCL^38,55,56^ as a matrix toughener,^3,4,27−35^ suggesting a potentially similar action mechanism in contrasting delamination. Indeed, provided PEO miscibility with the hosting epoxy resin, the matrix toughening mechanism should occur.

PEO nanofibrous mats were produced via an electrospinning process and then thermally and mechanically characterized before interleaving during CFRP lamination. The nano-modified composite was tested for evaluating the interlaminar fracture toughness in Mode I and Mode II loadings [double cantilever beam (DCB) and end-notched flexure (ENF) tests, respectively]. Flexural mechanical properties were assessed by quasi-static three-point bending (3PB) tests. Moreover, overall laminate thermomechanical properties were evaluated via dynamic mechanical analysis (DMA).

The work aims to demonstrate the feasibility of using PEO nanofibrous mats as a toughener in epoxy CFRP laminates, resulting in the first reported investigation of PEO application in the field of composite materials.

## 2 Materials and Methods

### 2.1 Materials

PEO (*M*_w_, 100,000 Da), chloroform (CHCl_3_), and acetone were purchased from Sigma-Aldrich and used without any preliminary treatment or purification. The prepreg used for composite production was a plain-weave carbon fabric, 200 g/m^2^, impregnated with epoxy matrix (GG204P IMP503Z-HT, G. Angeloni S.r.l., Venezia, Italy). The resin fraction is 42% on a volume basis, as stated by the technical datasheet.

### 2.2 PEO Electrospinning and Nano-Modified Laminates Production

PEO solution at 15% wt was prepared in CHCl_3_/acetone 60:40 wt (e.g., 1.5 g of polymer in 5.4 mL of CHCl_3_ and 6.9 mL of acetone) under magnetic stirring at room temperature until the formation of a homogeneous solution.

The PEO nanofibrous mat was produced using a four-needle electrospinning machine (Spinbow) equipped with 5 mL syringes. Needles (internal diameter, 0.51 mm; length, 55 mm) were joined to syringes via Teflon tubing. Nanofibers were collected on a 50 rpm rotating drum (tangential speed 0.39 m/s), covered with a poly(ethylene)-coated paper. The mat has the final dimensions of approximately 30 × 40 cm and a thickness in the 35–40 μm range, measured using an analogue indicator under a 360 g/m^2^ pressure. Under this measuring condition (for soft materials, such as nanofibrous membranes, thickness is dependent on the applied measuring pressure; for further information, refer to ref (57)), such thickness corresponds to a mat grammage of 12.2 ± 0.8 g/m^2^.

Electrospinning was carried out in an ambient atmosphere, at 23–26 °C, and 22–25% relative humidity. Process parameters were as follows: flow rate, 0.60 mL/h; electric potential, 19 kV; distance, 13 cm; and electrostatic field, 1.5 kV/cm.

CFRP panels for DCB, ENF, and DMA tests were produced via hand lay-up in an air-conditioned room (21–23 °C, 25–27% relative humidity). The nanofibrous membranes were directly applied with their paper substrate onto the prepreg during the hand lay-up. Before adding the next prepreg ply, the supporting paper was removed. To promote the impregnation of the nanofibrous mat, uncured panels underwent a preliminary treatment for 2 h at 40 °C under vacuum before curing. Then, they were cured in an autoclave for 2 h at 135 °C under vacuum with 6 bar of external pressure and a heating/cooling ramp of 2 °C/min.

Reference panels without nanofibrous mats were also produced for the sake of comparison. For details regarding panels and specimens dimensions, refer to the Supporting Information.

### 2.3 Characterization of the PEO Nanofibrous Mat and CFRP Laminates

The nanofibrous mat morphology was assessed via scanning electron microscopy (SEM, Phenom ProX). The thermal properties were investigated via differential scanning calorimetry (DSC) and thermogravimetric analysis (TGA). Tensile testing of the electrospun membrane was performed to evaluate the mechanical behavior.

DSC measurements were carried out on a TA Instruments Q2000 DSC modulated apparatus equipped with a refrigerated cooling system (RCS). The PEO nanofibrous mat sample (10 mg) was heated from −85 to 120 °C at a rate of 20 °C/min in a nitrogen atmosphere. PCL and Nylon 66 DSC thermograms were obtained at the same heating rate according to the procedures reported in refs (38) and (57).

The degree of crystallinity (χ_c_) was calculated according to the well-known equation
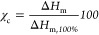
1where Δ*H*_m_ is the melting enthalpy of the sample and Δ*H*_m,100%_ is the melting enthalpy of a theoretical 100% crystalline polymer. Δ*H*_m,100%_ for PEO is 203–205 J/g.^58,59^

TGA (TA Instruments Q600) was carried out in an air atmosphere by heating the sample (10 mg) at a rate of 20 °C/min from 25 to 600 °C.

Tensile tests of nanofibrous mats were carried out using a universal testing machine (Remet TC-10) equipped with a 10 N load cell at a 10 mm/min crosshead separation rate. Nanofibrous mat specimen dimensions were 20 × 45 mm, width and gage length, respectively, prepared as previously reported.^57,63^ Standard load normalization based on the specimen cross-sectional area leads to inaccurate stress values due to the variability in determining the membrane thickness, which is affected by both the mat porosity and applied measurement pressure. Thus, tensile test data were normalized using a reliable method put forward by the authors, based on the specimen mass normalization of the load instead of its cross-sectional area,^57^ according to the following equation
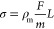
2where ρ_m_ is the material density (polymer density, not the apparent membrane density), *m* is the specimen mass, *L* is the specimen initial length, *F* is the force, and σ is the stress. For PEO, ρ_m_ is 1.125 g/cm^3^, as reported in the technical datasheet. By expressing ρ_m_ in mg/mm^3^, *F* in N, *m* in mg, and *L* in mm, σ is in MPa.

CFRP laminates were tested under Mode I and Mode II loadings for evaluating delamination resistance and via DMA to characterize their overall thermomechanical behavior.

DCB tests were performed for evaluating the energy release rate in Mode I loading (*G*_I_), both at the initial and propagation stages (*G*_I,C_ and *G*_I,R_, respectively), using the following equation^60^
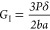
3where *P* is the load, δ is the crosshead displacement, *b* is the specimen width, and *a* is the crack length. DCB specimens were tested under a 3.0 mm/min crosshead separation rate.

ENF tests were carried out for evaluating the interlaminar fracture toughness in Mode II loading (*G*_II_), both at the initial and propagation stages (*G*_II,C_ and *G*_II,R_, respectively), using the following equation^61^
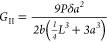
4where *L* is the span length between supports. ENF tests were carried out using a 3PB geometry with a 100 mm span (*L*) between supports; the specimen delamination length (*a*_0_) was set at 30 mm.

For each sample/test combination, three repetitions were done. *G*_R_ was evaluated considering a crack length range of 47–90 mm for Mode I and a 31–43 mm range for Mode II tests. Crack path micrographs were recorded using a Zeiss optical microscope, while delamination surfaces were investigated using a scanning electron microscope (SEM, Phenom ProX).

Washings of DCB delamination surfaces were carried out in distilled water at room temperature (25 °C) and at 85 °C for 6 h.

3PB tests were carried out using a 1 kN load cell, at a 5 mm/min rate, with a support span of 80 mm (support span-to-specimen depth ratios of 32:1), according to the reference standard.^62^

DMA (Netzsch DMA 242 E Artemis) was performed in the 3PB deformation mode using a 40 mm fixed span support. DMA was carried out from −80 to 250 °C at a 3 °C/min heating rate, 1 Hz frequency, an amplitude of 20 μm, and static force/dynamic force ratio = 1.5.

## 3 Results and Discussion

**Figure 1:**
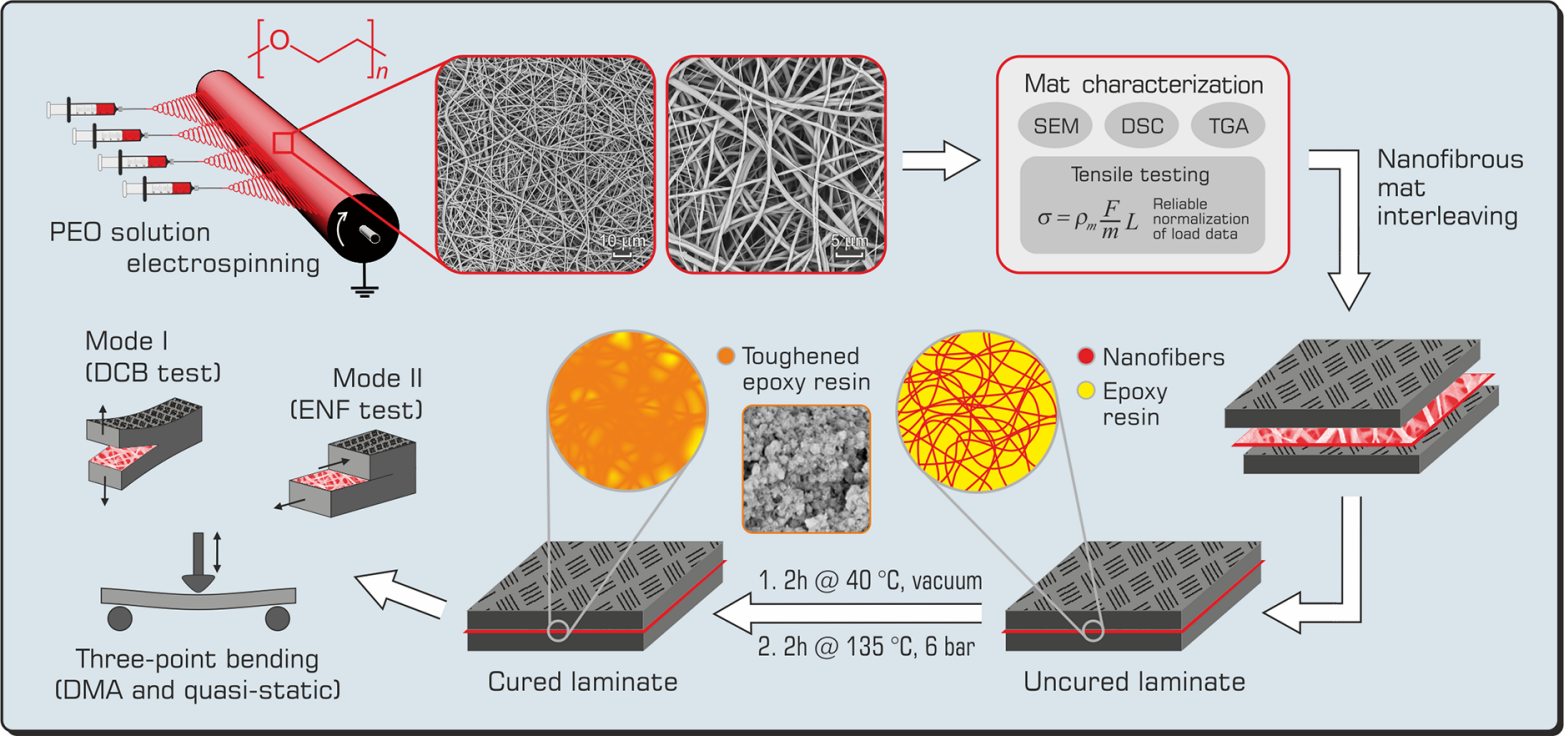
Overview of the work: PEO electrospinning and nanofibrous mat interleaving during lamination, curing, and testing of the nano-modified laminate with PEO nanofibers.

Figure 1 shows an overview of the entire work. The electrospun PEO, in the form of nanofibers, was used for the first time for modifying CFRP laminates by interleaving nanofibrous mats during the lamination step. After curing, the CFRP laminates, both modified and unmodified ones, were tested for evaluating the interlaminar and flexural properties in addition to the thermomechanical performance.

### 3.1 PEO Nanofibrous Mat Characterization

The electrospun PEO nanofibrous mat, shown in Figure 1, is constituted of randomly oriented nanofibers characterized by an average diameter of 503 ± 174 nm.

**Figure 2:**
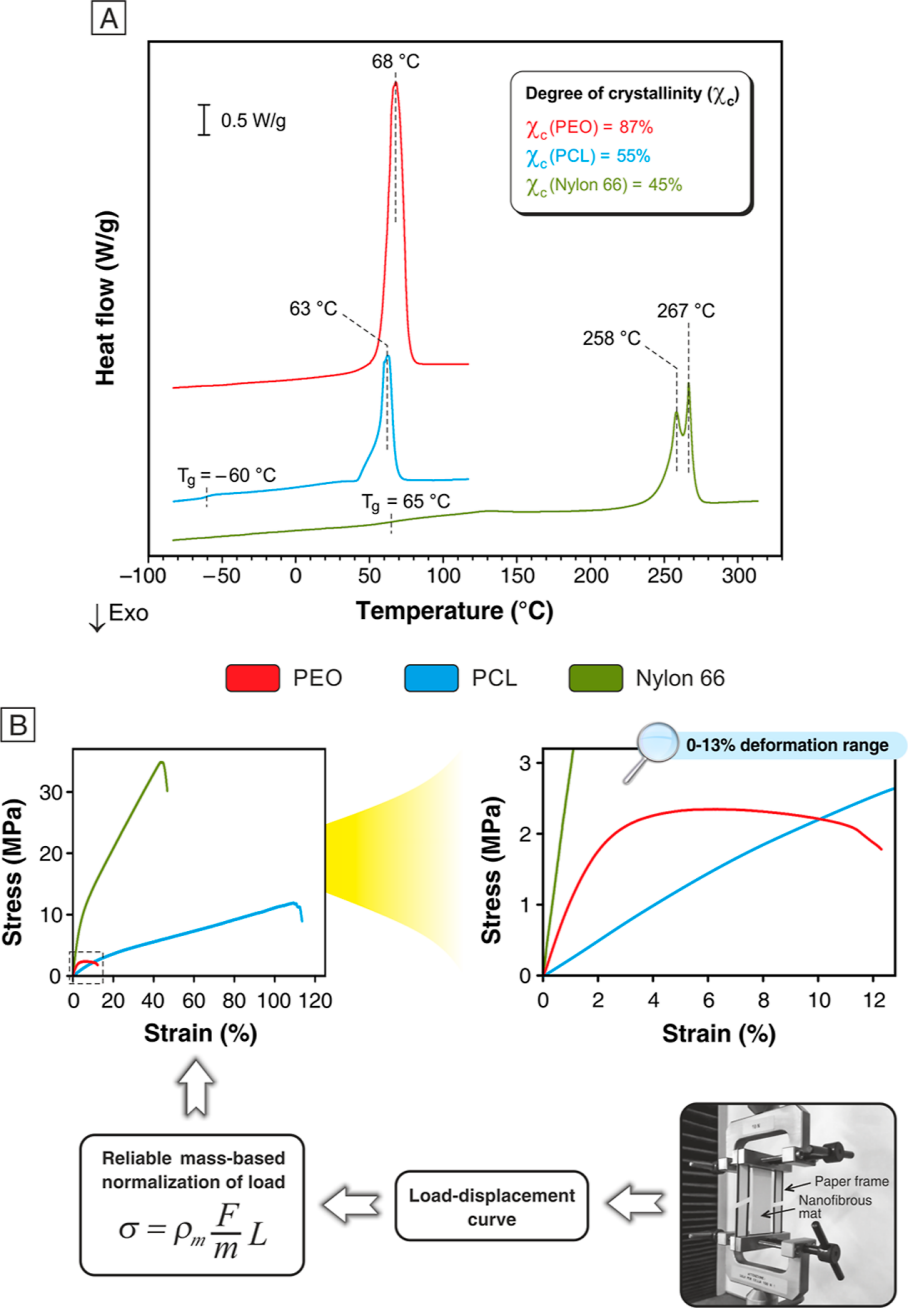
Thermal and mechanical characterization of nanofibrous mats: (A) DSC thermograms and (B) tensile stress–strain curves of PEO nanofibers. For comparison purposes, thermal and tensile behaviors of Nylon 66^57^ and PCL^38^ nanofibrous mats are also displayed.

As anticipated in the Introduction, the polyether PEO has almost the same thermal properties as the polyester PCL (Figure 2A): the glass transition temperature is well below the room temperature (≈−70 °C, not visible in the PEO thermogram, value taken from the literature^55^), and the melting temperature is near 70 °C. The melting behavior, highly reminiscent of the PCL one, suggests a potentially similar action at contrasting delamination. Indeed, provided PEO miscibility with the hosting epoxy resin, the matrix toughening mechanism should occur, as expected for epoxy-miscible polymers that melt or “fluidize” during the curing cycle, such as PCL and uncrosslinked rubbers.^39^

DSC analysis (Figure 2A) reveals a high degree of crystallinity (χ_c_ = 87%) of PEO nanofibers; as a consequence, the amorphous fraction is so limited that the glass transition is indeed not detectable. In the present case, the electrospinning process does not hamper the development of an extensive crystal phase, as may happen in semicrystalline polymers,^64,65^ due to the rapid solvent evaporation occurring during the fiber formation.

The tensile test highlights the fragile behavior of the PEO nonwoven, compared to mats made of Nylon 66 and PCL: the elongation at break (ε@σ_max_) is very limited, as well as the strength (σ_max_), while the elastic modulus (*E*) is relatively high (Figure 2B and Table 1). The PEO mat stiffness in combination with the very low mat properties at break (σ_max_ and ε@σ_max_) originates from the high PEO χ_c_, which is significantly higher than that of PCL and Nylon 66 nanofibers.

**Table 1:** Tensile Properties of the PEO Nanofibrous Mat Compared with Those of Thermoplastic Polymers Commonly Used as Interleaves for Enhancing Composite Interlaminar Fracture Toughness

nanofibrous mat	elastic modulus, *E* (MPa)	maximum stress, σ_max_ (MPa)	elongation at break, ε@σ_max_ (%)	toughness, *U* (J/cm^3^)
PEO	105 ± 4	2.4 ± 0.1	12 ± 1	0.24 ± 0.02
PCL^38^	35 ± 3	12 ± 2	112 ± 16	7.2 ± 1.4
Nylon 66^57^	296 ± 28	35 ± 3	46 ± 6	9.4 ± 1.3

The comparison between the different membrane types is reliable, thanks to the load normalization based on the mat specimen mass instead of its cross-sectional area, as previously demonstrated.^57^ Also, the membrane toughness (*U*) is extremely low compared to the other two nonwovens. However, it is worth pointing out that the very low mat properties at break (σ_max_ and ε@σ_max_), besides an extremely limited toughness, are not indicative of a poor reinforcing ability in the composite interlaminar region when the matrix toughening mechanism is envisaged, as in the case of PEO nanofibers.

Despite the fragile behavior, the PEO membrane is handleable and self-standing, allowing its simple integration into the composite laminate during the lamination step.

### 3.2 Thermal Stability of PEO Nanofibers and Laminate Curing

**Figure 3:**
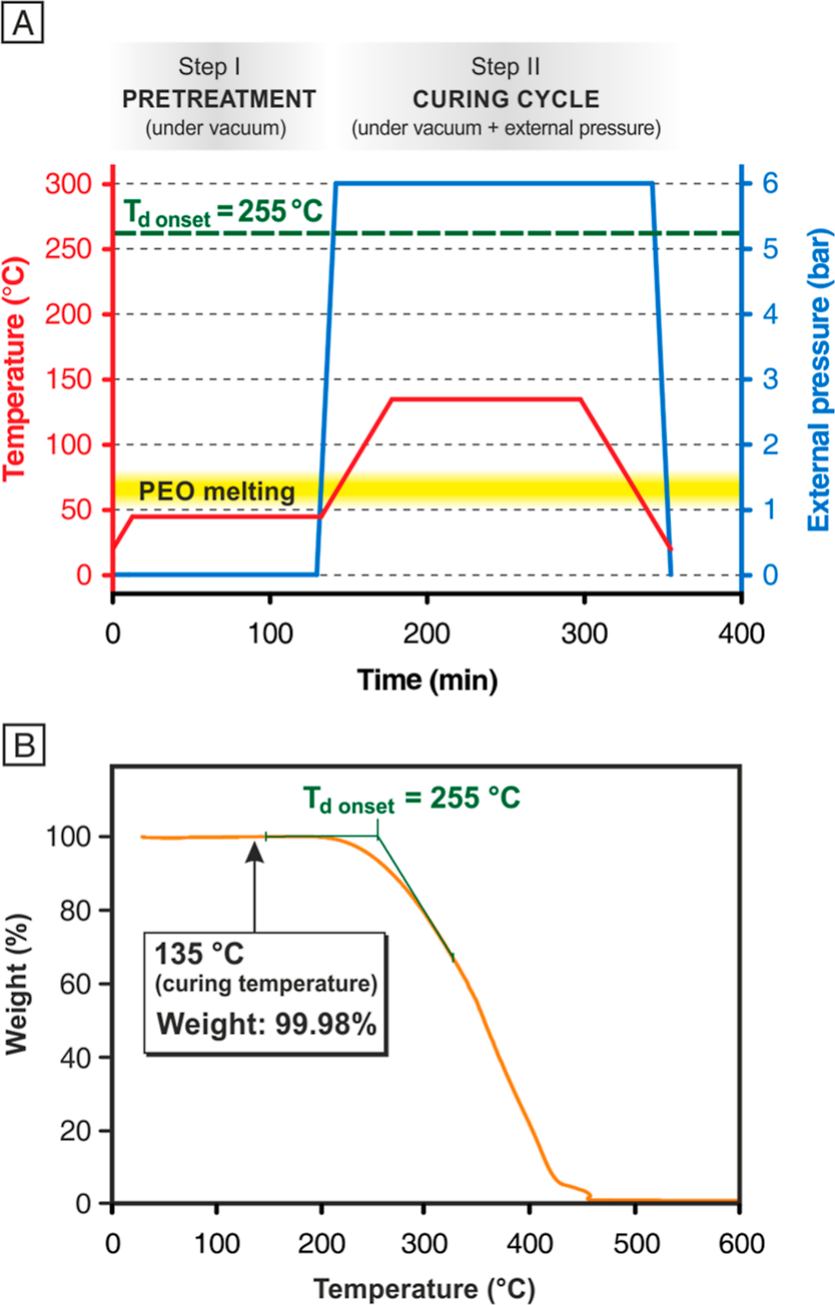
(A) Pretreatment and curing cycle steps used for CFRP laminates: temperature (red) and pressure (blue) profiles vs time. For better clarity, the *T*_d_ onset and melting temperature range of PEO nanofibers are also displayed. (B) TGA thermogram of the nanofibrous PEO membrane carried out in an air atmosphere.

Thermosetting matrices, such as epoxy resins, need to be cured under a certain combination of time and temperature. Here, the laminates were cured at 135 °C for 2 h, in accordance with the prepreg technical datasheet. Figure 3A shows the overall thermal treatment that CFRP panels underwent for their curing.

A preliminary TGA of the PEO membrane was carried out to ensure that the nanoreinforcement was stable at the curing cycle temperature. The thermo-oxidative degradation profile (Figure 3B) shows a degradation onset (*T*_d_) at 255 °C, well above the curing cycle temperature (135 °C), at which the weight loss is only 0.02%. Moreover, notwithstanding the extremely high specific surface area that the polymer possesses in the nanofibrous mat, TGA does not show any weight loss at low temperature that could be attributed to some extent of water absorption. Thus, PEO usage in composites is “safe” from a thermal point of view. Before the actual curing cycle (step II), a preliminary treatment at a low temperature (step I) was added for favoring the nanofibrous mat impregnation. During step I, only vacuum was applied (without external pressure) to prevent a high compaction of the nanofibrous mat, while the temperature was set below the PEO melting range (50–80 °C, as assessed via DSC, Figure 2A, and highlighted in yellow in Figure 3A) to avoid the nanostructure collapse prior to impregnation with the composite epoxy matrix.

### 3.3 Mode I and Mode II Interlaminar Fracture Toughness Evaluation

**Figure 4:**
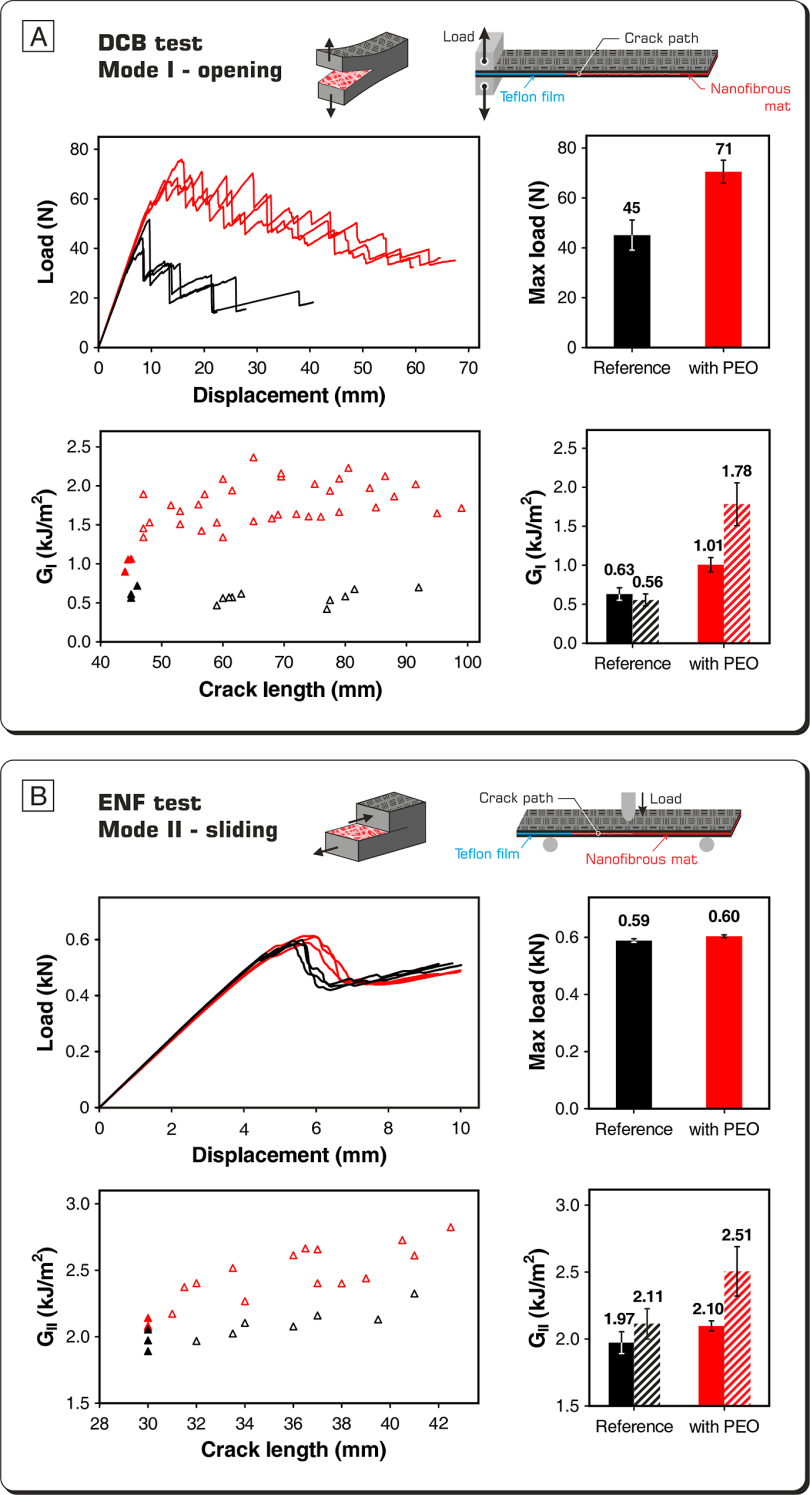
DCB (A) and ENF (B) tests results: load-displacement curves, maximum loads, *R*-curves, and average *G* (solid bars: *G*_C_, dashed bars: *G*_R_). Curves, points, and bars in graphs are represented in black for the reference CFRP and in red for the nano-modified one. In *G*_I_ and *G*_II_ graphs, solid triangles correspond to the first crack advancement and the empty ones to subsequent propagations.

The reinforcing effect of PEO nanofibers against the detrimental delamination phenomenon was assessed by performing DCB and ENF tests, thus evaluating the interlaminar fracture toughness in Mode I and Mode II loadings, respectively. These tests examine the specimen in different ways: in Mode I, the specimen beams are subjected to a perpendicular load with respect to the crack propagation plane, while in Mode II, a bending deformation is imposed to simulate the sliding of the two constituent beams. Taking into account the grammage of the interleaved PEO mat and the resin fraction of the CFRP prepreg, it is possible to estimate that the percentage of PEO in the interlaminar region of the central interface is between 7 and 8% wt. Figure 4 summarizes DCB and ENF test results.

The efficacy of PEO nanofibers in hindering delamination in Mode I loading is evident by simply analyzing the load-displacement curves, which give a preliminary overview of the laminate delamination resistance. The PEO-interleaved laminate curve displays a similar trend and slope to the unmodified CFRP until the first load drop, which appears to be significantly postponed. Moreover, load-displacement curves of the nano-modified composite present a more jagged profile, indicating that many subsequent crack advancements occur; besides, all of them are positioned at considerably higher load values in the diagram. The comparison of the maximum load recorded during the DCB test shows a +58% in average for the nano-modified laminate, suggesting a substantial effect of PEO on the delamination behavior.

The performance gap between the PEO-modified laminate and the reference CFRP is confirmed by the *R*-curves (*G*_I_ vs crack length). The increase in the energy release rate (*G*), both at the initial (*G*_I,C_) and propagation (*G*_I,R_) stages, is significant. Regarding the first crack advancement, *G*_I,C_, the nano-modified laminate performs 60% better than the reference one. In propagation, *G*_I,R_, the performance enhancement is even higher: +221%. Moreover, while the energy release rate for the crack propagation in the reference laminate drops slightly after the initial event, the nano-modified material displays a strong increase in *G*_I,R_. The latter behavior makes the material intrinsically safer with respect to the unmodified counterpart since even if a crack accidentally starts, its propagation is nonetheless hampered.

Regarding the Mode II delamination performance, the action of PEO nanofibers is more limited. The *G*_II,*C*_ enhancement is 7%, while a higher improvement is found in G_II,R_, where the energy release rate increases by 19%.

Comparing the present results with literature data,^3,4,33,34,39^ two main statements can be made as follows: (i) PEO nanofibers perform generally better than common PCL and polyamide (Nylon 6 and Nylon 66) nanofibrous mats under Mode I delamination and (ii) under Mode II loading, the PEO-modified laminate exhibits a limited *G*_II_ enhancement; however, it is in line with some reported results. Regarding the Mode I delamination, it can be affirmed that in almost all the presented cases, the *G*_I_ enhancement is in the range of 20–50%, with a few reported cases showing better or worse results. Therefore, the *G*_I_ improvement provided by PEO nanofibers (up to 221%) is sensibly higher than the one delivered by most PCL and Nylon membranes. In the cited literature, only one work claims a *G*_I_ enhancement of 92% when PCL nanofibers are integrated, while the others report a maximum improvement of 60%. Given that the thermal properties of PEO and PCL are almost the same (Figure 2A), the high PEO efficacy at hindering Mode I delamination suggests a relevant role of the polymer chemistry in matrix toughening. The cited literature data for Mode II delamination are more contrasting. Some works report a good *G*_II_ enhancement in the range of 50–80%, and a few others report a better one or even no nanofiber effect. However, there are also several studies reporting a *G*_II_ improvement in the 7–30% range, showing results similar to the ones obtained with the PEO nanofibers. It can be concluded that PEO modification performs better than PCL one under Mode I loading, though it shares with PCL the mechanism action through matrix toughening.

### 3.4 Crack Path and Delamination Surface Analyses

**Figure 5:**
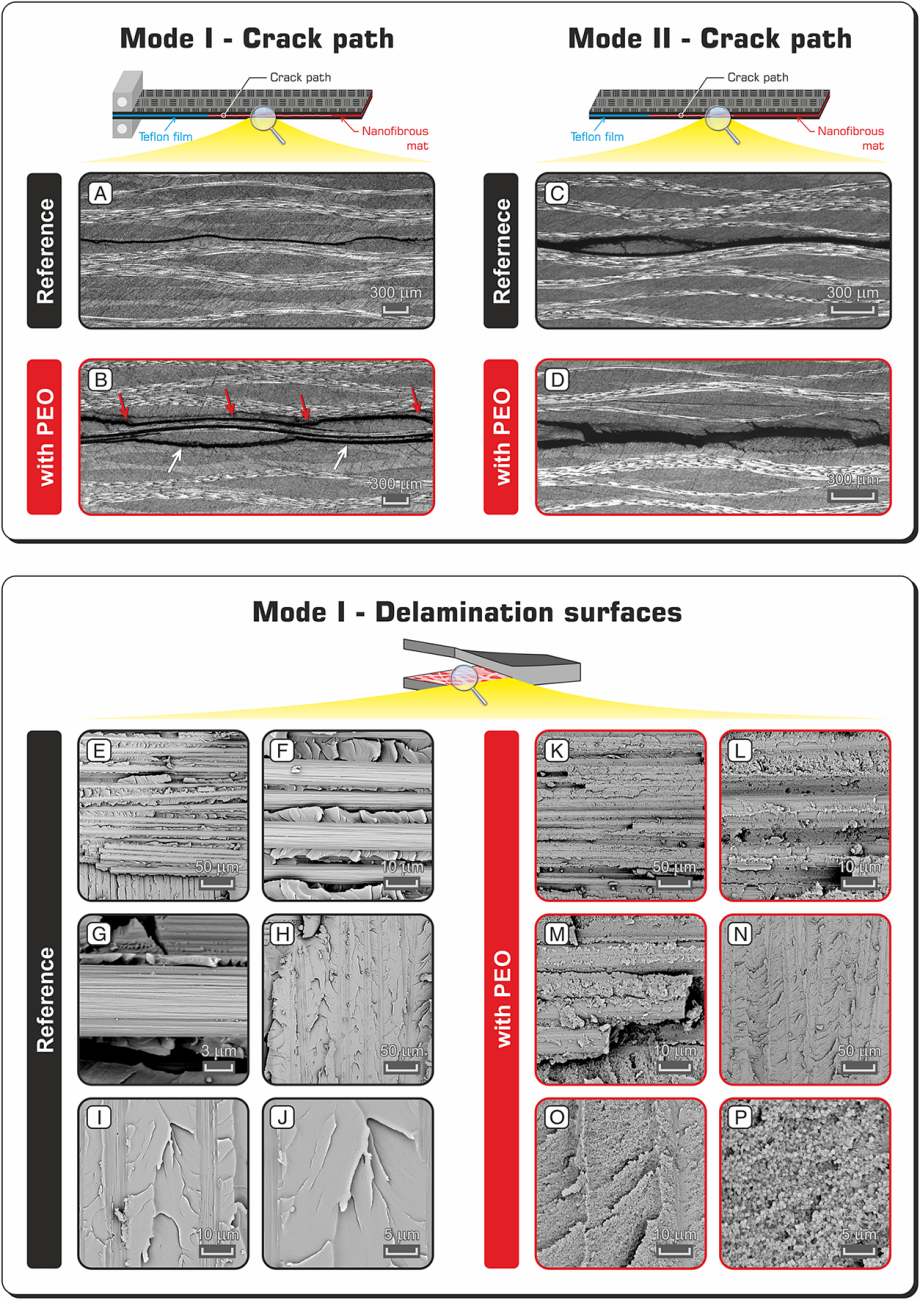
Top (A–D) images of DCB specimens after Mode I and Mode II delamination tests. Red arrows indicate the designed crack plane (central plane) reinforced with PEO nanofibers and white arrows indicate the plane adjacent to the central one. Down (E–P) images showing delamination surfaces after DCB tests.

The crack path analysis of the nano-modified laminate after the DCB test reveals a strong toughening action of PEO (Figure 5B). Indeed, the crack paths are uneven, and they have more planes than the PEO-modified central one. Instead, the reference CFRP laminate displays a regular crack path that propagates along the central plane only (Figure 5A).

The different aspect of crack paths is in accordance with the discussed load-displacement profiles and the calculated *R*-curves, confirming the strong effect of PEO nanofibers on increasing the interlaminar fracture toughness. Also, the ENF crack path of the PEO-modified CFRP (Figure 5D) evidences that higher damages occurred during the crack advancement. However, this behavior does not correspond to a strong *G*_II_ enhancement, as displayed in Figure 4B.

Regarding Mode I, a similar behavior was found for CFRPs reinforced with rubbery nanofibers made of NBR mixed with Nomex^25^ or PCL:^39^ the toughening action was so effective that multiple crack paths and even a carbon fabric break occurred. In the latter work, PCL-only nanofibers were tested too, revealing a low attitude for increasing the interlaminar properties. In the present case, PEO strongly affects the delamination behavior, suggesting a relevant role of the polymer chemistry upon matrix toughening, which is not only a consequence of the mere polymer thermal properties (they are almost the same for PEO and PCL, Figure 2A). Because PEO nanofibers melt in the 50–80 °C temperature range (onset and endset of the endotherm melting peak, Figure 2A), well below the curing cycle temperature of 135 °C, the thermoplastic polymer is expected to act via matrix toughening. Indeed, PEO can be mixed with the epoxy resin, which is plasticized, leading to an increased interlaminar fracture toughness.

The analysis of the delamination surfaces after DCB tests is helpful to understand the matrix toughening extent (Figure 5E–P). The unmodified CFRP shows the matrix arrayed in wide flat planes, typical of an epoxy brittle fracture. The surface morphology of the PEO-modified laminate is completely different: the sharp and smooth matrix planes are replaced by a rougher surface. Extensive phase separation can be detected by deeply analyzing the surface morphology: it is entirely disseminated by irregular spheres, having an average diameter of 389 ± 68 nm. In literature, phase separation phenomena in similar cases are reported;^66−68^ however, their occurrence is not mandatory when dealing with thermoplastic materials that melt below the curing cycle temperature. For example, our previous work regarding NBR/PCL blend nanofibrous mat interleaving^39^ reveals that neither rubbery nanofibers nor PCL ones give phase separation, while a bulk NBR film promotes it. Therefore, the attained interface morphology depends not only on the electrospun polymer but also on the combination of the nanofiber material and matrix type.

**Figure 6:**
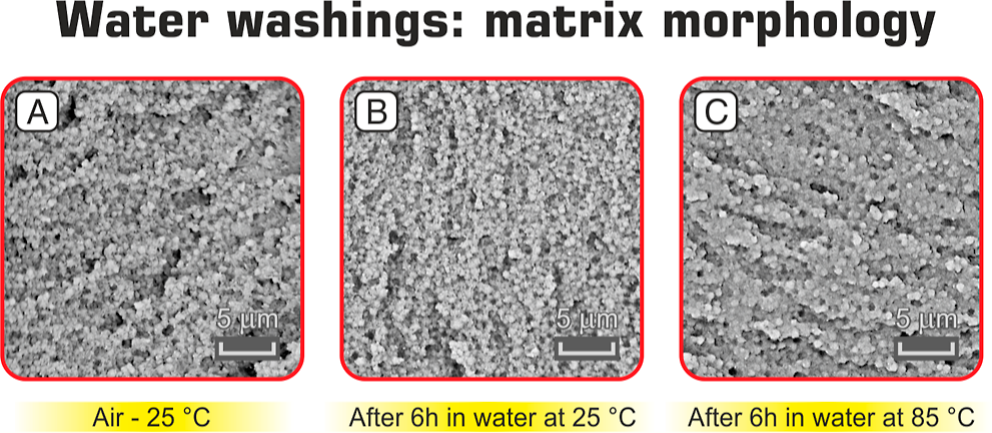
Matrix morphology of Mode I delamination surfaces: as is (A) and after washings in water (B,C).

Since PEO is a water-soluble polymer, its rich phase-separated regions could represent a potential point of weakness in the nano-modified CFRP under ambient conditions where humidity is always present. For this reason, DCB delamination surfaces were subjected to washings in water. SEM images in Figure 6 show that no significant morphological variation occurs even when the sample is treated with hot water at 85 °C, a temperature above the PEO melting temperature (≈70 °C). This result probably accounts for some “coating” of the spheres with the hosting epoxy resin, preventing PEO dissolution. Such a behavior is very encouraging because it should guarantee sufficient reliability for the use of interleaved PEO nanofibers in composites operating under real ambient conditions. Moreover, if the nanomodification does not occur until the laminate edges, the potential water sorption will be the same as that of the unmodified composite.

### 3.5 Flexural Mechanical Properties of the PEO-Modified CFRP

**Figure 7:**
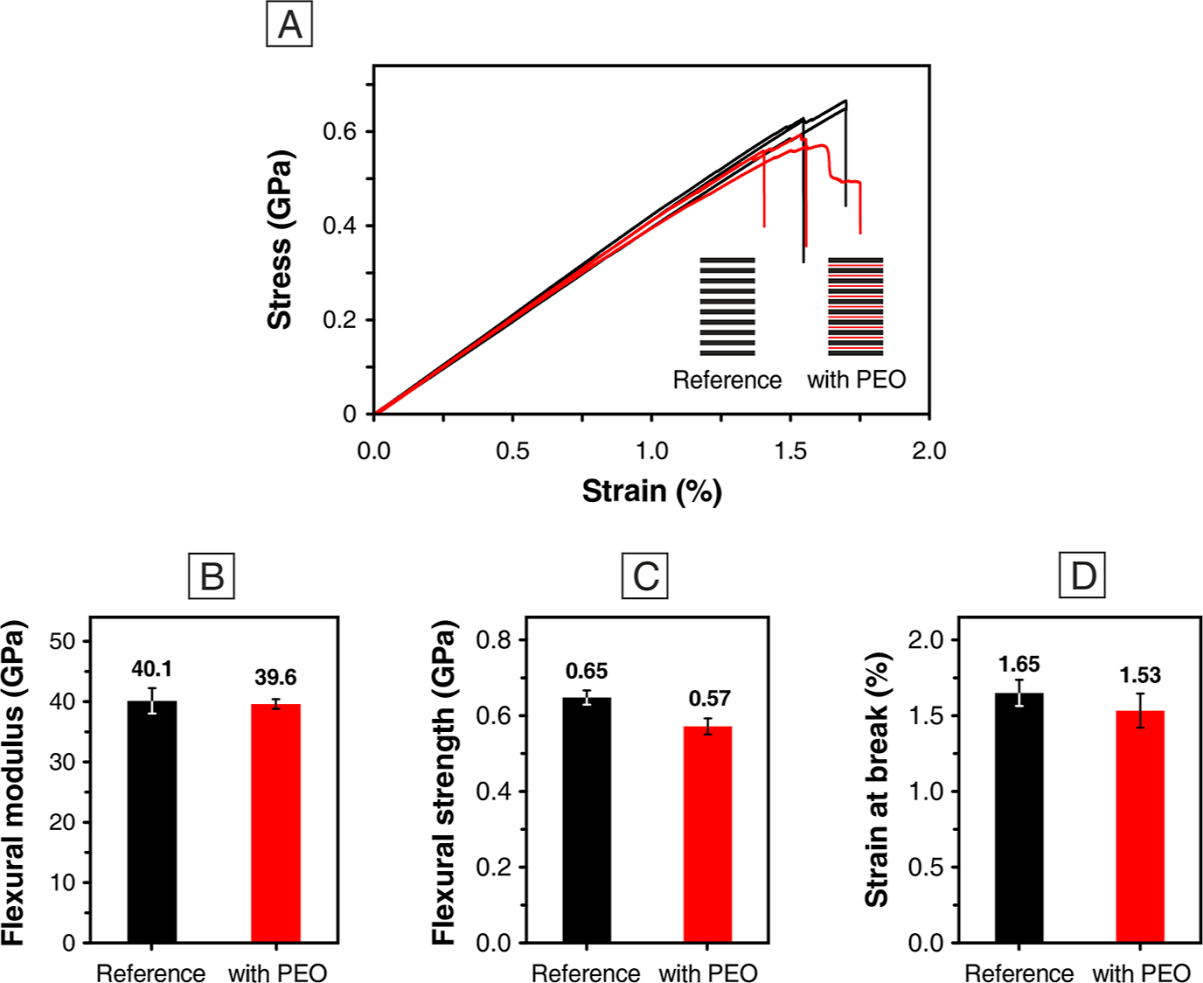
3PB tests of CFRP laminates: (A) stress–strain curves (reference CFRP given in black and the nano-modified one in red) and histograms of the (B) flexural modulus, (C) flexural strength, and (D) strain at break.

Stiffness lowering is one of the most critical side effects that may affect laminates modified with soft materials, such as nanofibrous nonwovens made of low thermal and/or mechanical properties. Although PEO does not possess a high mechanical performance nor high thermal properties, 3PB tests reveal that composite mechanical properties are almost unaffected by PEO addition (Figure 7). In fact, despite the extensive PEO nanomodification (all the CFRP interfaces were modified to emphasize the effect of the nanofiber integration), the original laminate stiffness is fully maintained, as well as the strain at break, while only the flexural strength is slightly reduced (−12% of the mean value).

### 3.6 Thermomechanical Properties of the PEO-Modified CFRP

**Figure 8:**
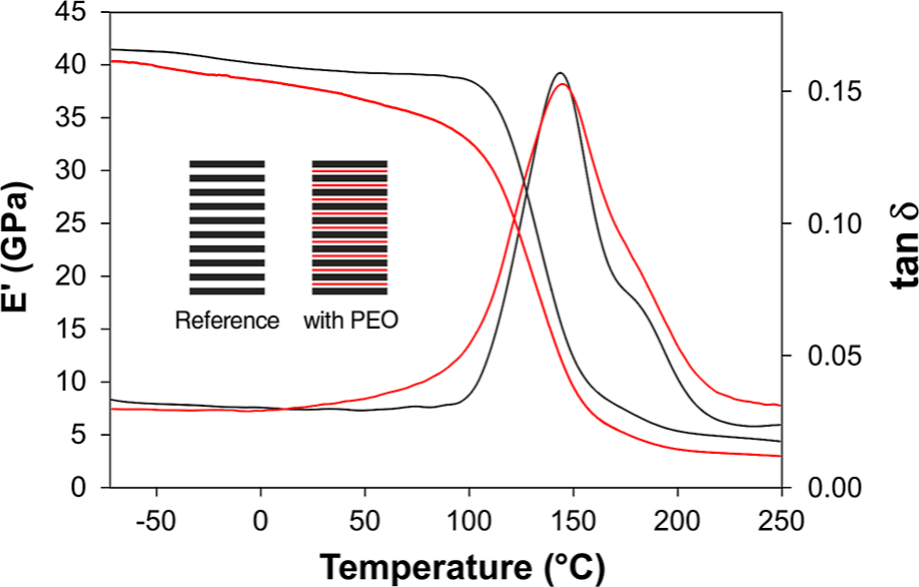
DMA tests: storage modulus (E′) and tanδ representative curves of the reference CFRP (black) and of the laminate with all the interfaces nanomodified (red).

Another critical aspect, besides the cited stiffness reduction, affecting CFRP nanomodification is the potential *T*_g_ lowering. Evaluating the overall thermomechanical laminate behavior is fundamental to know the possible material application field. Figure 8 shows DMA of the PEO-modified laminate in comparison with that of the unmodified CFRP. *T*_g_, evaluated as the onset of *E′* lowering, stays close to that of the reference CFRP, showing only a slight reduction (106 °C vs 113 °C). The storage modulus (*E′*) trend of the PEO-modified laminate is slightly lowered with respect to that of the reference CFRP one, while the tanδ peak is almost the same as that of the reference CFRP. However, it can be safely assumed that the mechanical behavior of both samples should be similar (at least at low temperatures), considering the results found by quasi-static 3PB tests, showing no significant flexural modulus reduction (Figure 7B). It is worth underlining that all nine laminate interfaces were modified for maximizing the PEO effect on the laminate thermomechanical properties, leading to an overall PEO fraction in the resin + thermoplastic mixture of about 7% wt (≈3% wt of the whole nano-modified laminate weight). However, in real situations, it is not necessary to toughen all the interfaces but only the one(s) most critical or the regions where the stress is concentrated the most, such as free edges, holes, and ply-drops,^69^ thus reducing the impact of the nanomodification.

## 4 Conclusions

This work demonstrates the feasibility of using PEO nanofibrous mats as an effective epoxy toughener in high-performance CFRP laminates, resulting in the first reported investigation of PEO application in the field of composite materials. The thermoplastic polymer can be easily inserted locally during the lamination step as a nanofibrous membrane produced via electrospinning. Nano-modified composites revealed a significant ability of PEO nanofibers at contrasting Mode I delamination (DCB test), showing +60% and +221% in *G*_I,C_ and *G*_I,R_, respectively. The high toughening action delivered by PEO is also confirmed by the crack path and delamination surface analyses. In particular, the latter reveals that strong epoxy matrix toughening occurred with phase separation phenomena. The efficacy of the PEO membrane under Mode II loading (ENF test) is still present, though to a lower extent (+7% in *G*_II,C_ and +19% in *G*_II,R_).

Mechanical properties are practically unchanged upon extensive nanomodification, as 3PB tests proved, and DMA reveals that the laminate *T*_g_ is close to that of the reference CFRP (106 °C vs 113 °C). Moreover, the toughened matrix surface seems to be unaffected by water washings: it should guarantee a sufficient reliability in the use of interleaved PEO nanofibers in composites operating under real ambient conditions.

## Supporting Information Available

Details on the production and characterization of CFRP laminates (PDF)
