# Supplementary material for: New Application Field of Polyethylene Oxide: PEO Nanofibers as Epoxy Toughener for Effective CFRP Delamination Resistance Improvement
Source: ACS Omega. 2022 Jun 24;7(27):23189–200. doi: 10.1021/acsomega.2c01189 (PMC9281329; doi:10.1021/acsomega.2c01189)
Supplement: Supplementary file 1 — ao2c01189_si_001.pdf [file ao2c01189_si_001.pdf]

Supporting Information

New application field of polyethylene oxide: PEO nanofibers as epoxy toughener for effective CFRP delamination resistance improvement

*Emanuele Maccaferri[a, *], Jacopo Ortolani[a,b], Laura Mazzocchetti[a,b, *], Tiziana Benelli[a,b], Tommaso Maria Brugo[b,c], Andrea Zucchelli[b,c], Loris Giorgini[a,b]*

[a] *Department of Industrial Chemistry "Toso Montanari", University of Bologna, Viale Risorgimento 4, 40136 Bologna, Italy.*
[b] *Interdepartmental Center for Industrial Research on Advanced Applications in Mechanical Engineering and Materials Technology, CIRI-MAM, University of Bologna, Viale Risorgimento 2, 40136 Bologna, Italy.*
[c] *Department of Industrial Engineering, University of Bologna, Viale Risorgimento 2, 40136 Bologna, Italy.*

* Corresponding authors: emanuele.maccaferri3@unibo.it, laura.mazzocchetti@unibo.it

S1. Production of CFRP laminates for DCB, ENF, 3PB and DMA tests

CFRP panels for DCB, ENF, 3PB and DMA tests were produced via hand lay-up in an air-conditioned room (21-23 °C, 25-27% relative humidity). The nanofibrous membranes were directly applied with their paper substrate onto the prepreg during the hand lay-up. Before the addition of the next prepreg ply, the supporting paper was removed. To favour the impregnation of the nanofibrous mat, uncured panels underwent a preliminary treatment of 2 h at 40 °C under vacuum before curing cycle in autoclave. Then, they were cured in an autoclave (2 h at 135 °C, under vacuum, 6 bar external pressure, heating/cooling ramp 2 °C/min).

CFRP panels for DCB and ENF tests (Figure S1), 140 × 190 mm, are constituted by 14 CFRP plies in total. Only the central interface was modified with nanofibrous PEO. A Teflon film was used as a crack trigger. The specimens were obtained by cutting out the panel; edge parts (minimum 15 mm) were discarded for avoiding any inhomogeneity.

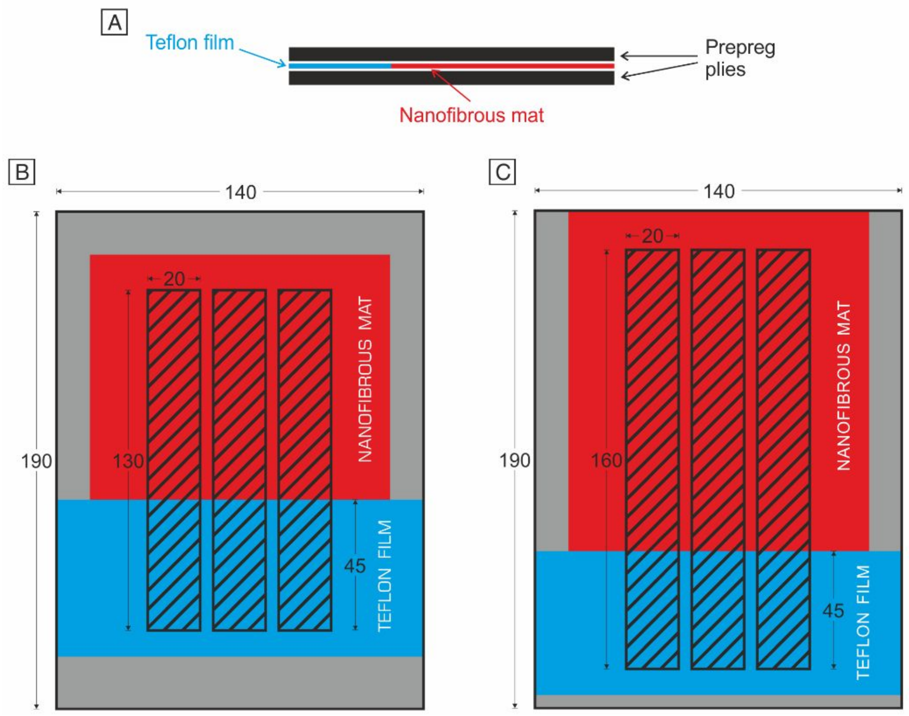

Figure S1. CFRP panels for characterization of delamination resistance: laminate section view (A) and dimensions (in mm) of panels and specimens for DCB (B) and ENF (C) tests.

DCB specimens have the following final dimensions: 130 mm total length, 20 mm width (b), 45 mm initial crack length (a_0). To anchor the specimen on the testing machine, aluminium blocks were fixed to the DCB specimen with epoxy resin glue.

ENF specimens had the following dimensions: 160 mm total length, 20 mm width (b), 45 mm initial crack length (a_0).

The laminates for the 3PB and DMA tests are constituted by 10 CFRP plies in total. The PEO-modified sample has all the interfaces nanomodified (Figure S2). The specimens for the 3PB test are 110 × 10 mm, obtained by cutting out 150 × 70 mm CFRP panels; the ones for DMA are 50 × 8 mm, obtained by

cutting out 80 × 70 mm CFRP panels. In both cases, edge parts of the panel (≈ 15 mm) were discarded to ensure specimen homogeneity.

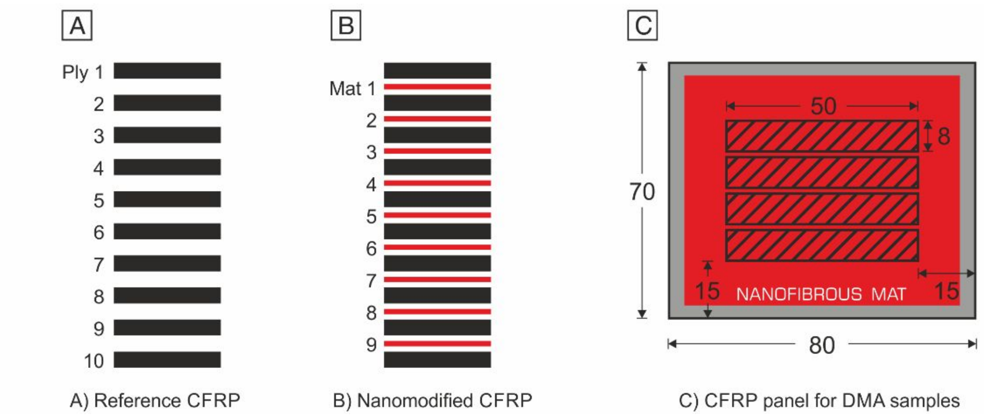

Figure S2. Stacking sequence of plies/mats for production of 3PB and DMA laminates: (A) unmodified and (B) nanomodified CFRP laminates. (C) Dimensions (in mm) of CFRP panels and specimens for DMA.
